# America's red gold: multiple lineages of cultivated cochineal in Mexico

**DOI:** 10.1002/ece3.1398

**Published:** 2015-01-08

**Authors:** Michael G Campana, Nelly M Robles García, Noreen Tuross

**Affiliations:** 1 Department of Human Evolutionary Biology, Harvard University11 Divinity Avenue, Cambridge, Massachusetts, 02138; 2 Proyecto Conjunto Monumental de AtzompaCalle Reforma 501, esq. Constitución. Sala IV. Centro Histórico, Oaxaca, Oaxaca, 68000, Mexico

**Keywords:** Cochineal, genomics, Mexico, Peru, phylogeography

## Abstract

Cultivated cochineal (*Dactylopius coccus*) produces carminic acid, a valuable red dye used to color textiles, cosmetics, and food. Extant native *D. coccus* is largely restricted to two populations in the Mexican and the Andean highlands, although the insect's ultimate center of domestication remains unclear. Moreover, due to Mexican *D. coccus* cultivation's near demise during the 19th century, the genetic diversity of current cochineal stock is unknown. Through genomic sequencing, we identified two divergent *D. coccus* populations in highland Mexico: one unique to Mexico and another that was more closely related to extant Andean cochineal. Relic diversity is preserved in the crops of small-scale Mexican cochineal farmers. Conversely, larger-scale commercial producers are cultivating the Andean-like cochineal, which may reflect clandestine 20th century importation.

## Introduction

Domesticated cochineal (*Dactylopius coccus*) is a New-World scale insect cultivated for carminic acid, a potent scarlet dye used to color textiles, cosmetics, and food (Chávez-Moreno et al. 2009). With the use of mordants and adjuncts, carminic acid dyes produce colors ranging from pinks to deep purples and black (Phipps 2010). From the conquest of the Aztec Empire by the Spanish until the advent of laboratory-synthesized colorants in the 19th century, cochineal dye was the preeminent source of scarlet coloring. Cochineal was one of the primary exports from New Spain (after gold and silver) and played a critical role in the highland Mexican economy, where commercial production was centered (Chávez-Moreno et al. 2009). Cochineal dye's monetary value was so high that its production was a Spanish state secret and pre-Columbian codices describing its use were destroyed to prevent piracy. After the development of artificial red dyes, cochineal production nearly disappeared, including from highland Mexico. Since the 1970s, cochineal production has started to resurge due to the discovery of carcinogenic and hazardous properties of many synthesized dyes (Chávez-Moreno et al. 2009).

Cochineal insects (*Dactylopius* spp.) are endemic American phytophagous scale insects of the monogeneric family Dactylopiidae. Ten species are currently recognized (Van Dam and May 2012), although highly divergent biotypes within individual species have been identified, suggesting possible cryptic speciation (Mathenge et al. 2009). Four wild species are endemic to north and central Mexico (*D*. *confusus*,*D. gracilipilus*,*D. opuntiae*, and *D. tomentosus*), while an additional five wild species (*D. austrinus*,*D. ceylonicus*,*D. confertus*,*D. salmianus*, and *D. zimmermanni*) are endemic to South America (Rodríguez et al. 2001; Chávez-Moreno et al. 2009; Van Dam and May 2012). As an antimicrobial and antipredatory defense mechanism, all cochineal insects (both wild and cultivated species) synthesize the anthraquinone carminic acid. Of the *Dactylopius* species, domesticated *D. coccus* produces the most carminic acid (∽20% of dry body weight) (Wouters and Verhecken 1989; Chávez-Moreno et al. 2009). Additionally, *D. coccus* lacks the protective waxy coating that the wild forms possess, making it more susceptible to both weather fluctuations and predation (Chávez-Moreno et al. 2009).

Cochineal insects are obligate parasites of cacti (primarily *Opuntia* spp.), with individual *Dactylopius* species/biotypes preferring different host cactus species. *D*. *coccus* can survive on a wide range of host cactus species. While cultivated insects are primarily raised on domesticated nopal (*Opuntia ficus-indica*), *D. coccus* can also parasitize *Nopalea cochenillifera* and numerous *Opuntia* species including *O. atropes*,*O. crassa*,*O. fuliginosa*,*O. hyptiacantha*,*O. jaliscana*,*O. megacantha*,*O. pilifera*,*O. robusta*,*O*. *streptacantha*,*O*. *tomentosa*, and *O*. *undulata* (Rodríguez et al. 2001; Chávez-Moreno et al. 2011). *D. coccus* competes with other *Dactylopius* species for these hosts across its range, although some other species also parasitize cactus species not utilized by *D. coccus* (e.g., *Cylindropuntia* spp.) (Chávez-Moreno et al. 2011).

**Figure 1:**
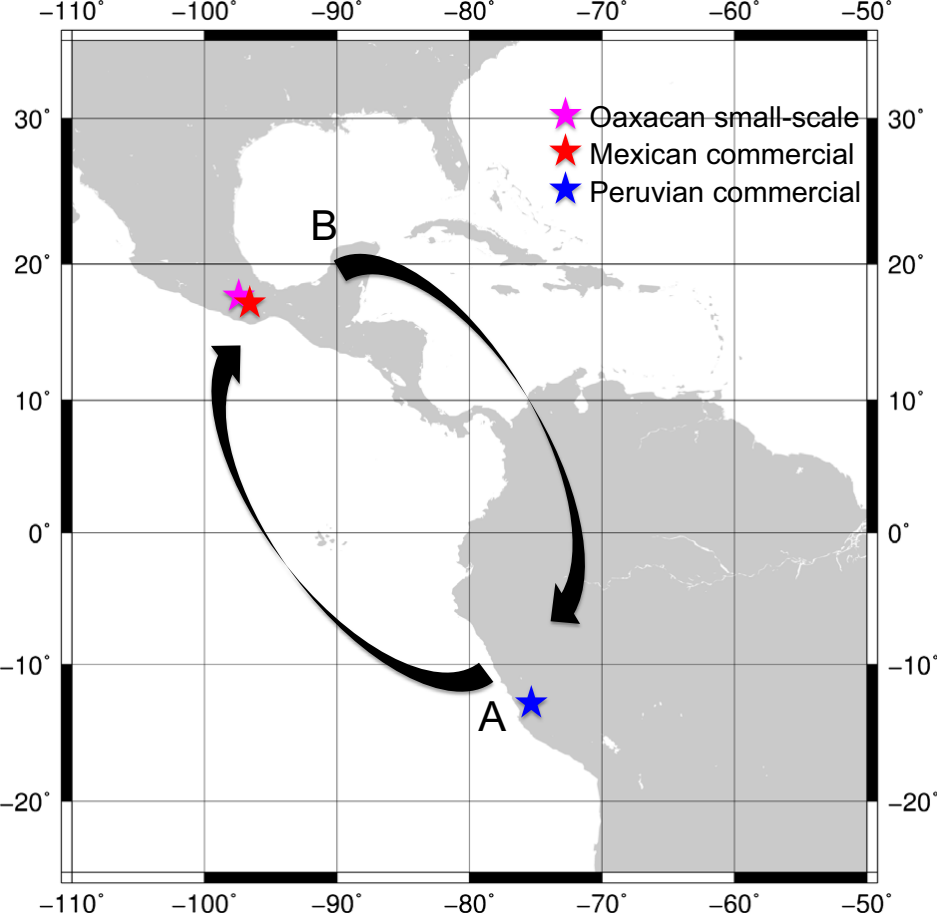
Map depicting the competing *Dactylopius coccus* origin hypotheses: (A) *D. coccus* originated in Peru and subsequently spread to Mexico; (B) *D. coccus* evolved in Mexico and was later introduced to Peru, possibly after domestication. *D*. *coccus* sampling locations for the genomic analyses are also shown.

The geographic origin of domesticated cochineal is debated (Fig.1). “Native” populations are located in highland Mexico (centered in Oaxaca state, but also found in Puebla, Tlaxcala, and the Valley of Mexico) and in the Andes of southern Peru (Chávez-Moreno et al. 2009; de Ávila Blomberg 2005; Rodríguez et al. 2001). Feral populations have also been reported in neighboring Chile. Coccidoculture was successfully introduced to Spain, the Canary Islands, Argentina, Guatemala, and South Africa during the 19th and 20th centuries. This disjunct distribution is unexpected as cochineal species have limited dispersion capability: female cochineals are sessile, attaching themselves to the host plant immediately after hatching, while males are winged, but die quickly after fertilizing females, surviving only approximately three days in their adult form (de Ávila Blomberg 2005). Furthermore, although its host *Opuntia* species can thrive in multiple ecological zones, *D. coccus* is limited to arid and semi-arid habitats (Chávez-Moreno et al. 2009).

*D. coccus*'s dispersed geographical pattern raises the question of whether the current day distribution is natural or the result of deliberate introduction of the insects in prehistory. The earliest known cochineal-dyed textiles were discovered in Paracas, Peru (10th to 12th century AD), but the first evidence of cochineal farming was found in Mexican Toltec (10th century AD) sites (Rodríguez et al. 2001; Chávez-Moreno et al. 2009). Based on a phylogenetic analysis of morphological characters, Rodríguez et al. (2001) argued for a South American origin. Additionally, Mexican *D. coccus* is reliant on human propagation and protection for survival, while Andean insects survive ferally (Ramírez-Puebla et al. 2010). Conversely, de Ávila Blomberg (2005) argued that the presence of eight species that prey on domesticated cochineal in Mexico, as opposed to only one extant species in the Andes, indicates a Mexican origin. Genetic evidence is lacking: before this project, only 58 short DNA sequences (<800 bp each) were available for the entire *Dactylopius* genus.

Although genetic analyses could clarify the history of domesticated cochineal, they require phylogenetically informative variation to exist in extant populations. Whether extant Mexican cochineal exhibits such variation is unclear. While Oaxaca, Mexico was once the center of cochineal production, the Oaxacan cochineal industry nearly disappeared during the 19th century (Chávez-Moreno et al. 2009). Cochineal crops were deliberately destroyed during the Mexican War of Independence. The industry never recovered due to the competition from foreign production and the development of synthetic dyes. This bottleneck may have greatly reduced the level of diversity. Furthermore, Mexican populations may have become introgressed with Peruvian stocks during the 20th century (Chávez-Moreno et al. 2009). After the destruction of the Oaxacan cochineal industry, the center of production shifted to Peru. As the majority of Mexican *D. coccus* crops had gone extinct, some Mexican farmers may have been forced to obtain Peruvian stocks to start production. Trade of *D. coccus* stocks with the Canary Islands has also been noted in Mexico (Chávez-Moreno et al. 2009), although this is less likely to obscure phylogeographic information since the Canary Island population was introduced from Mexico around 1825 A.D. (Piña Luján 1980). Here, we assess the level of extant diversity of Mexican *D. coccus* through analysis of mitochondrial genetic markers and *de novo* whole-genomic sequencing.

## Materials and Methods

### Cochineal sample collection

Grana (dried female cochineal used for dye production) and fresh *D. coccus* females were obtained from small-scale farmers and large-scale commercial vendors in Mexico, Chile, and Peru (Table1). As large-scale commercial vendors may conglomerate crops from different farmers in each year, we tested multiple crop years from several producers (Table1). We also obtained historic grana of unknown provenance from the Peabody Museum of Archaeology and Ethnology (Harvard University) to evaluate whether extinct diversity might be preserved in historic specimens. Additionally, we collected wild female cochineal (*Dactylopius* spp.) by hand in Oaxaca, Mexico, for comparison with the cultivated species.

**Table 1:** Single-insect samples collected and analyzed for mitochondrial markers. The geographic and/or commercial source of the material as well as year of collection is given for each sample. Also noted is whether the sample was obtained from a small-scale cochineal farmer (“Small-scale”), a large-scale commercial vendor (“Commercial”), or wild-caught (“Wild”). “Sample Type” states whether the sample was derived from grana or fresh insects. The total sample size and the number of sequenced cytochrome c oxidase I (*cox1*) and 12S rRNA mitochondrial genes are also given

Sample	Source	Year	Cultivation type	Sample type	*N*	*cox1*	12S rRNA
Oaxaca1	Oaxaca, Mexico	2012	Small-scale	Fresh	20	14	0
Oaxaca2	Oaxaca, Mexico	2010	Small-scale	Grana	5	0	0
Oaxaca3	Oaxaca, Mexico	2010	Small-scale	Grana	5	4	0
Oaxaca4	Oaxaca, Mexico	2010	Small-scale	Grana	5	0	0
Oaxaca5	Oaxaca, Mexico	2010	Small-scale	Grana	5	0	0
Mexico1	Mexico (textile store “Teotitlan,” Oaxaca)	2011	Commercial	Grana	10	10	10
Mexico2	Mexico (textile store “Teotitlan,” Oaxaca)	2012	Commercial	Grana	5	4	0
Peru1	Peru (wildcolours.org.uk)	2011	Commercial	Grana	10	10	10
Peru2	Peru (aurorasilk.com)	2011	Commercial	Grana	10	5	10
Peru3	Peru (La Tierra Dye Co.)	2011	Commercial	Grana	10	10	0
Peru4	Peru (aurorasilk.com)	2012	Commercial	Grana	10	10	0
Chile1	Chile (aurorasilk.com)	2011	Commercial	Grana	10	0	0
Chile2	Chile (aurorasilk.com)	2012	Commercial	Grana	10	1	0
Museum	No provenance (Peabody Museum)	Unknown	Unknown	Grana	10	0	0
OaxacaWild	Oaxaca, Mexico	2012	Wild	Fresh	41	25	0

### Mitochondrial marker analyses

**Figure 2:**
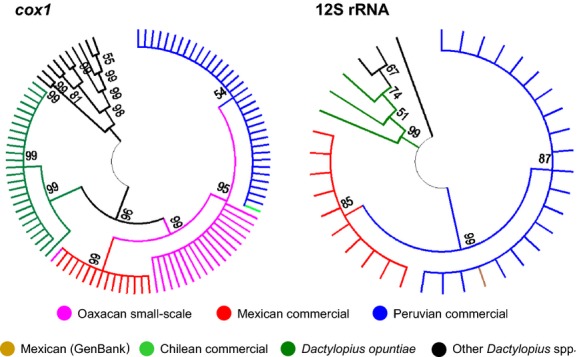
Condensed maximum-likelihood trees of *Dactylopius coccus* cytochrome c oxidase I (*cox1*) and 12S rRNA mitochondrial genes. Topology robustness was tested with 100 bootstrap replicates.

DNA was extracted from 166 single insects using the PowerSoil kit (MO BIO, Carlsbad, California, USA) and the QIAamp® DNA Mini Kit (Qiagen, Valencia, California, USA) according to manufacturer's instructions. The dataset included 40 insects cultivated by small-scale Oaxacan farmers, 75 from large-scale commercial producers (15 Mexican, 40 Peruvian, and 20 Chilean), 10 historic grana samples without provenance, and 41 wild *Dactylopius* from Oaxaca (Table1). The mitochondrial cytochrome c oxidase I (*cox1*) and 12S rRNA genes were amplified by the polymerase chain reaction and dideoxy-terminator sequenced (Appendix). The 12S rRNA experiments were omitted for most individuals as we found only three single-nucleotide polymorphisms in an initial subset of 30 individuals (10 Mexican and 20 Peruvian grana from commercial vendors), and the results were in agreement with the more informative *cox1* results (Table1; Fig.2). The obtained sequences were compared with 11 *cox1* (representing *D. opuntiae* [*n *=* *1] and *D. tomentosus* [*n *=* *10]) and seven 12S rRNA sequences (including *D. opuntiae* [*n *=* *3], Mexican *D. coccus* [*n *=* *1], *D. confusus* [*n *=* *1], *D. ceylonicus* [*n *=* *1], and *D. tomentosus* [*n *=* *1]) obtained from GenBank. While this sample is not representative of the entire *Dactylopius* genus, it includes all publicly available data for these genes.

### Whole-genome sequencing library construction

Due to the discovery of limited mitochondrial variation, we conducted whole-genomic sequencing on *Dactylopius coccus* to better understand domesticated cochineal phylogenies. Three bulk extracts representing cochineal raised by Oaxacan small-scale farmers or sold by Mexican and Peruvian commercial vendors were subjected to Pool-Seq (Schlötterer et al. 2014; Fig.1). Bulk DNA was extracted from 50 individuals each (Schlötterer et al. 2014; Appendix). Sequencing libraries were prepared from the bulk extracts using the PrepX Illumina Kit (IntegenX, Pleasanton, California, USA) and NEXTflex™ DNA Barcodes (Bioo Scientific, Austin, Texas, USA) on the Apollo 324 robotic platform (IntegenX). Paired-end 150-bp sequences were generated on one-quarter of an Illumina HiSeq 2500 lane. A total of 5.2–5.5 million paired sequences were obtained per library.

### Identification and phylogenetic analysis of genomic sequence variants

A draft *Dactylopius coccus* genome assembly was constructed using JR-Assembler 1.02 (Chu et al. 2013; Table2). The final assembly was 18.6 Mbp long with an N50 of 378,999 bp (Table2). The quality-controlled merged sequence reads were aligned against the *D. coccus* assembly using BWA 0.7.5 (Li and Durbin 2009, 2010) in order to identify sequence variants. A total of 1.99 Gbp of reads (106.8× mean depth) were aligned to the assembly. Analysis of the assembly using BEDTools 2.17.0 (Quinlan and Hall 2010), however, showed significant variation in coverage across the genome and between samples (per sample mean depth ± standard deviation: 9.4× ± 78.7×, 43.3× ± 26.9×, and 54.0× ± 34.0× for the Oaxacan small-scale farm, Mexican commercial, and Peruvian commercial samples, respectively). Genotypes were called using SAMtools 0.1.19 (Li et al. 2009).

**Table 2:** *Dactylopius coccus* genome assembly statistics

Assembly length	18,613,147 bp	Mean sequencing depth	106.8×
N50	378,999 bp	L50 count	12
No. scaffolds	1499	Mean scaffold length	12,417 bp
Maximum scaffold length	1,388,629 bp	Minimum scaffold length	200 bp
Genome %A	20.89%	Genome %T	20.97%
Genome %G	29.02%	Genome %C	29.12%

### Selection on the *Dactylopius coccus* genome

To determine whether the cochineal genome was undergoing detectable natural or artificial selection, we predicted genic sequences using GeneMark-ES 2.3c (Borodovsky and Lomsadze 2011). The ratio of nonsynonymous to synonymous (N/S) SNPs was calculated using SnpEff 3.6a (Cingolani et al. 2012). Tajima's *D* was calculated using 500-bp windows with VCFtools 1.0.9 (Danecek et al. 2011).

## Results

### Mitochondrial DNA analyses

The grana accessions' DNA preservation varied, probably due to different procedures used for preparation (e.g., boiling and air drying). We were unable to obtain sequences for all individuals due to the variation in DNA preservation. We obtained 68 *cox1* (18 from Oaxacan small-scale farmers, 14 from commercial Mexican vendors, 35 from commercial Peruvian vendors, and 1 from Chilean commercial vendors) and 30 12S rRNA *Dactylopius coccus* sequences (10 Mexican and 20 Peruvian insects from commercial vendors) (Table1; Fig.2). We sequenced 25 wild Oaxacan cochineal *cox1* genes. All the Oaxacan wild cochineal we collected clustered with *Dactylopius opuntiae* (Fig.2).

We observed nine credible substitutions in 1003 bp of *D. coccus* mitochondrial DNA (0.90% divergence): six substitutions in 559 bp of *cox1* sequence (1.1% divergence) and three substitutions in 454 bp of 12S rRNA (0.66% divergence). We identified three *cox1* and two 12S rRNA *D. coccus* haplotypes (Fig.2). Peruvian commercial cochineal *cox1* sequences differed by one substitution from the Oaxacan small-scale farm insect specimens. A third divergent *cox1* haplotype (an additional five substitutions) was found in Mexican commercial samples. The Chilean sample clustered with the Peruvian commercial grana. The 12S rRNA tree resolved the same two major clades (Peruvian commercial/Oaxacan small-scale farm insects versus Mexican commercial cochineal).

### Genomic SNP phylogenetic analyses

**Figure 3:**
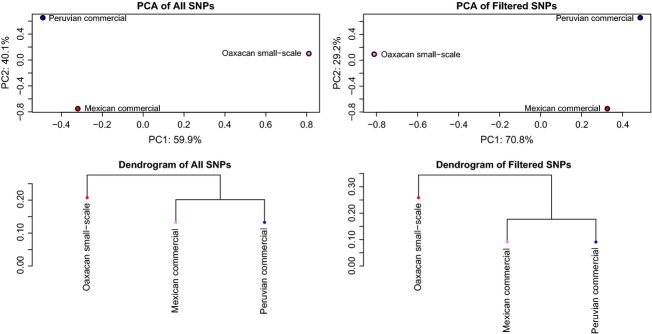
Relatedness between Oaxacan small-scale farm, Mexican commercial, and Peruvian commercial cochineal bulk samples. Principle component analysis (top row) separates the Oaxacan small-scale farm insects from the commercial specimens, with the first principle component explaining the majority of the variation (59.9% and 70.8% in the unfiltered and filtered SNP datasets, respectively). Identity-by-state analysis (bottom row) of these SNP datasets produces dendrograms with congruent topology.

A total of 11,517 genomic variants (including 10,598 polymorphic single-nucleotide polymorphisms [SNPs]) were identified in the three *D. coccus* pools. To account for sequencing errors, collapsed repetitive regions and apparent variants deriving from *D. coccus*-like environmental contaminants, we refined the SNP dataset by requiring that each site be sequenced a minimum depth of 5× per pool (15× total depth) and a maximum of 100× per pool (300× total depth). The refined SNP dataset included 82 high-confidence polymorphic SNPs (135× mean total sequencing depth). Both the raw and filtered SNP datasets were analyzed by principal component (PCA) and identity-by-state relatedness analyses using SNPRelate 0.9.12 (Zheng et al. 2012; Fig.3). While SNPRelate was designed to analyze individuals, no similar software is yet available for Pool-Seq data. To corroborate the SNPRelate results, we calculated genomic differentiation (mean *F*_ST_) of the informative sites using PoPoolation2 1.201 (Kofler et al. 2011) using the same SNP filtering criteria as in the SNPRelate analyses. Additionally, SNP-sharing analysis was performed on the raw SNP dataset using VCFtools 1.0.9 (Danecek et al. 2011).

**Figure 4:**
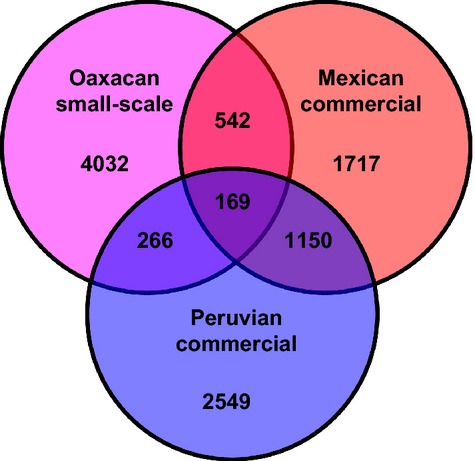
Venn diagram depicting numbers of genomic SNPs unique to and shared between each bulk *Dactylopius coccus* sample.

All genomic SNP analyses had congruent results (Fig.3; Table3). The first principal component separated the Oaxacan small-scale farm sample from the Mexican and Peruvian commercial vendor specimens. Similarly, in the identity-by-state relatedness analyses, the Mexican and Peruvian commercial samples form a clade, with the Oaxacan small-scale farm sample being more distantly related (Fig.3). Genomic differentiation analysis also separated the Oaxacan small-scale farm sample from the two commercial samples (Table3). Additionally, the commercial samples from Mexico and Peru share more SNPs with each other than either do with the Oaxacan small-scale farm sample (Fig.4). These results indicate that the Mexican and Peruvian commercial samples are more closely related to each other than they are to Oaxacan small-scale farm cochineal. Notably, both the genomic differentiation and the SNP-sharing analyses show that the Oaxacan small-scale farm sample is slightly closer related to the Mexican commercial cochineal than to the Peruvian cochineal (Table3; Fig.4). Unfortunately, we are unable to ascertain precise ages of these genomic clades as we have no paleontological calibration point and the most closely related sequenced genome, the pea aphid (*Acyrthosiphon pisum*), is too divergent to align against the *D. coccus* draft genome sequence (International Aphid Genomics Consortium 2010).

**Table 3:** Genomic differentiation between the three cochineal bulk samples. Values are listed as mean *F*_ST_ ± standard deviation

	Oaxacan small-scale	Mexican commercial	Peruvian commercial
Oaxacan small-scale		0.0842 ± 0.0901	0.1097 ± 0.0897
Mexican commercial	0.0842 ± 0.0901		0.0096 ± 0.0058
Peruvian commercial	0.1097 ± 0.0897	0.0096 ± 0.0058	

### Selection on the *Dactylopius coccus* genome

GeneMark-ES predicted 8003 genes. A total of 4245 SNPs were located in putative exonic regions, of which 3028 were nonsynonymous and 1217 were synonymous substitutions (combined N/S for all samples = 2.49). Although the N/S ratio was greater than one for all bulk samples (1.96, 2.73, and 2.73 for the Oaxacan small-scale farm, Mexican commercial, and Peruvian commercial samples, respectively), Tajima's *D* found no strong evidence of selection on the cochineal genome (mean absolute value of *D *± standard deviation: 0.0560 ± 0.260). Furthermore, there was no difference in selection effect between genic (0.0600 ± 0.271) and nongenic (0.0552 ± 0.258) regions of the genome (Student's *t*-test, *P *=* *0.2151), which suggests that the high N/S ratios are not associated with selection.

## Discussion

We find no effect on the mitochondrial DNA diversity that can be attributed solely to human management. Nevertheless, the *cox1* and 12S rRNA mitochondrial diversity is limited (three and two haplotypes, respectively) with one Mexican haplotype diverging from the other two, suggesting some form of bottleneck in the past. Nonfunctionally constrained mitochondrial markers (such as the control region) may be more variable. While it is tempting to attribute the observed bottleneck to human management, a more likely explanation is cytoplasmic incompatibility due to *Wolbachia* infection, a process that can produce false phylogeographic signal in arthropod phylogenetic trees (Hurst and Jiggins 2005). *Dactylopius* host numerous endosymbionts (Ramírez-Puebla et al. 2010), including the Alphaproteobacterium *Wolbachia* (Pankewitz et al. 2007). We detected *Wolbachia* sequences in both the single-marker and genomic analyses (Appendix). Furthermore, we found only one mitochondrial haplotype in the wild Oaxacan cochineal (*D. opuntiae*), suggesting that limited mitochondrial diversity is common across *Dactylopius* species.

Similarly, we found no conclusive evidence that the cochineal genome is under strong natural or artificial selection. Nevertheless, we observed only one *D. coccus* genomic sequence variant every ∽1600 nucleotides, which suggests a relatively slow mutation rate for insects (for comparison, *Drosophila simulans* has a SNP every ∽40 bases) (Begun et al. 2007; Hu et al. 2013). Further research is required to determine whether the slow mutation rate reflects selection.

The genomic phylogeny suggests that extant Mexican *D. coccus* derive from at least two source populations. One of these populations appears to be Mexican in origin, while the other is more closely related to Peruvian cochineal. Moreover, the distinctiveness between the “Mexican” and “Peruvian” clades suggests long-term isolation between the populations, which does not support the hypothesis of continuous and extensive trading of cochineal stocks during the pre-Columbian era as has been proposed previously (Chávez-Moreno et al. 2009). This observation supports contentions by local Mexican cochineal farmers that Peruvian stock may have been recently imported into Oaxaca with the renewed interest in cochineal production. However, our genomic differentiation and SNP-sharing results suggest that Mexican commercial cochineal may also have some local Mexican ancestry, even if it primarily derives from recently imported Peruvian stock.

Notably, the mitochondrial and genomic phylogenies are incongruent. The *cox1* tree clusters the Peruvian grana and Mexican fresh insect accessions, but the genomic SNP data indicate that the two grana samples form a clade. *Wolbachia* infection is a likely cause of the discrepancy between the mitochondrial DNA and the genomic variant phylogenies (Hurst and Jiggins 2005). Alternatively, this incongruence could reflect recent introgression (Zakharov et al. 2009), which would be consistent with recent importation of South American cochineal into Mexico.

Further genomic research is required to establish *D. coccus*'s domestication center(s) with confidence. Our cochineal dataset does not permit us to identify the ultimate source population. Additionally, although *Wolbachia* strains exhibit phylogenetic and phylogeographic patterning (Russell et al. 2009), we were unable to pinpoint the source location of *D. coccus* through sequencing and analysis of its *Wolbachia* endosymbiont (Appendix). Our results, however, show that phylogenetically informative variation survives in the crops of Oaxacan small-scale cochineal farmers. Nevertheless, future analyses will need to carefully control for the effects of recent clandestine Peruvian introgression into Mexican stocks.

## Figures and Tables

**Figure A1:**
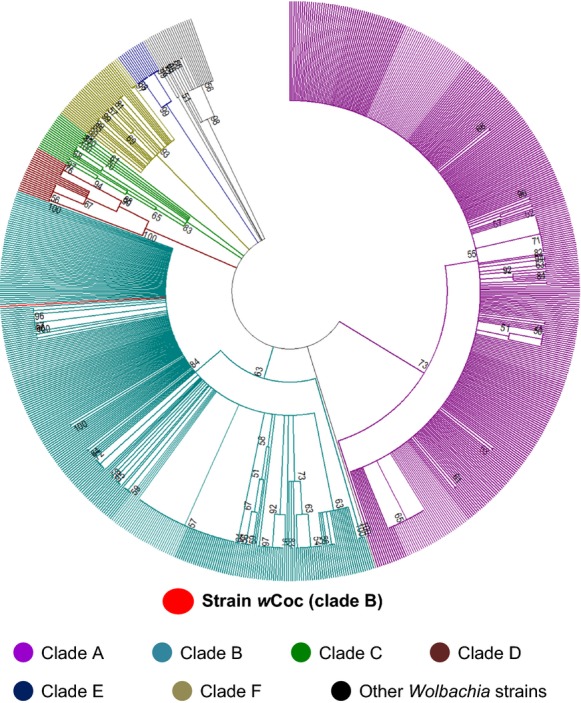
Condensed maximum-likelihood tree of 797 partial *Wolbachia ftsZ* genes. The *Wolbachia* endosymbiont of *Dactylopius coccus* (strain “*w*Coc”) falls in clade B. Clade nomenclature follows Lo et al. 2002. The tree was constructed under a Tamura–Nei substitution model with invariant sites and a gamma distribution for substitution rates (four gamma categories) and tested with 100 bootstrap replicates. Only clades supported by at least 50 replicates are noted.

## Data Accessibility

Sanger sequences have been deposited in GenBank (accessions KJ701865–KJ702008). Genome assemblies and associated sequence reads have been deposited in the BioProject archive (accession PRJNA244295).

## Appendix

### Single-marker Analyses

Based on the available cochineal and scale insect phylogenetic literature, we analyzed the mitochondrial cytochrome c oxidase I (*cox1*) and 12S rRNA genes and the nuclear 18S rRNA and elongation factor 1*α* (*EF1α*) genes (C.W. Mathenge, P. Holford, R. Spooner-Hart, G.A.C. Beattie, Unpublished data; Morse and Normark 2006; Ramírez-Puebla et al. 2010). DNA targets were amplified by PCR on an MJ Research PTC-200 DNA engine thermocycler. Each 25 *μ*L reaction contained 1× BIOLASE Diamond mix (Bioline, Taunton, Massachusetts, USA), 0.2–0.4 *μ*mol/L each primer and 3–5 *μ*L DNA. 12S and 18S reactions also contained 10 ng BSA. Primers are listed in TableA1. Final *cox1*, 12S rRNA, and 18S rRNA thermocycling programs consisted of an initial denaturation step at 95°C for 5 min (*cox1*, 12S rRNA) or 12 min (18S rRNA), 45 cycles of denaturation at 94°C for 30 sec, 50°C for 30 sec and 72°C for 45 sec, and a final extension step at 72°C for 10 min. The *EF1α* thermocycling program consisted of an initial denaturation step at 95°C for 4 min, 45 cycles of denaturation at 94°C for 30 sec, 50–55°C for 1 min and 72°C for 1 min, and a final extension step at 72°C for 4 min. PCR products were assessed by agarose gel electrophoresis. *EF1α* reactions produced multiple bands; therefore, the expected fragment of ∽1150 bp was isolated from the gel and purified using the QIAquick® Gel Extraction Kit (Qiagen). PCR products were treated with ExoSAP-IT™ (GE Healthcare) and then sequenced in both directions on an ABI 3730xl (Applied Biosystems) sequencer.

**Table A1:** PCR primers used to amplify *Dactylopius coccus* genetic markers

Marker	Forward primer	Reverse primer	Reference
12S rRNA	5′-AAGAGTGACGGGCRATTTGTACATA-3′	5′-GTGCCAGCAGTWGCGGTTA-3′	Ramírez-Puebla *et al*. (2010)
18S rRNA	5′-CTGGTTGATCCTGCCAGTAG-3′	5′-CCGCGGCTGCTGGCACCAGA-3′	Ramírez-Puebla et al. (2010)
*cox1*	5′-TCCGRATAGAACTWATAAAYACYAA-3′	5′-TAAACTTCAGGGTGACCAAAAAATCA-3′	C.W. Mathenge, P. Holford, R. Spooner-Hart, G.A.C. Beattie, Unpublished data
*EF1α*	5′-GATGCTCCGGGACAYAGA-3′	5′-ATGTGAGCGGTGTGGCAATCCAA-3′	Morse & Normark (2006)

Some initial *cox1* PCRs using the forward primer 5′-GGTCAACAAATCATAAAGATATTGG-3′ amplified sequences matching *Wolbachia* (Folmer et al. 1994). These contaminants were discarded. We observed no variation in the nuclear markers (13 *EF1α* and seven 18S rRNA sequences) consistent with their relatively slow rates of mutation. Therefore, these markers were not considered further. For the final *cox1* and 12S rRNA datasets, condensed maximum-likelihood trees were constructed with 100 bootstrap replicates under a Hasegawa–Kishino–Yano (Hasegawa et al. 1985) substitution model with invariant sites and a gamma distribution (four gamma categories) for substitutions in MEGA 6.06 (Tamura et al. 2013).

### Whole-genome Sequencing

Bulk extracts were constructed including 50 fresh insects or grana each. Fresh insects were digested using proteinase K in buffer ATL from the QIAamp® DNA Mini Kit (Qiagen) and ethanol-precipitated on site in Mexico. The precipitated DNA was transported dry back to the laboratory at Harvard where it was resuspended and purified using Econo-Pac 10DG columns (Bio-Rad). Grana were digested using buffers ATL (with proteinase K) and AL from the QIAamp® kit. Digested grana bulk extracts were vacuum-filtered and concentrated using Vivaspin® 15 30-kDa MWCO columns. Extracts were then exchanged into PCR-grade water and fractionated using Econo-Pac 10DG columns in order to separate the DNA from carminic acid. DNA-rich fractions were collected and purified using the QIAquick PCR Purification Kit (Qiagen).

Bulk extracts were sheared to ∽200 bp average length using a S220 Focused-Ultrasonicator (Covaris, Inc., Woburn, Massachusetts, USA). DNA-sequencing libraries were constructed using the PrepX Illumina Kit (IntegenX) and NEXTflex™ DNA Barcodes (Bioo Scientific) on the Apollo 324 robotic platform (IntegenX) according to the manufacturer's instructions. Libraries were quality-controlled via analysis on an Agilent 2100 using a high-sensitivity DNA chip and quantified using the KAPA Library Quantification Kit – Illumina/Universal (KAPA Biosystems) and a Qubit® Fluorometer. A total of 13 PCR cycles using the NEXTflex™ kit enriched the indexed libraries to sequenceable concentrations. PCR-enriched libraries were requantified and pooled in equimolar ratios. Paired-end 150-bp sequences were generated on one-quarter of an Illumina HiSeq 2500 lane.

After demultiplexing using CASAVA 1.8.2, mate-paired sequences were merged using PANDAseq 2.4.0 (Masella et al. 2012). Adapter artifacts were removed using TagDust 1.12 (Lassman et al. 2009). PCR duplicates were removed using CD-HIT 4.6 (Li et al. 2012). Final datasets were quality-controlled using FastQC 1.32 (Andrews n.d.).

A *Dactylopius coccus* genome assembly was constructed using JR-Assembler 1.02 from the original unpaired reads (Chu et al. 2013). The final four base pairs of each read were removed to improve sequence quality as recommended by Chu and colleagues (Chu et al. 2013). JR-Assembler uses complete reads to assemble the genome sequence via seed extension, which improves assembly of large genomes in comparison with de Bruijn graph assemblers such as SOAPdenovo2 (Luo et al. 2012) and ABySS (Simpson et al. 2009). We found that de Bruijn graph assemblers (SOAPdenovo2 and ABySS) produced unsatisfactory *D. coccus* assemblies, probably due to the relatively low sequencing depth and presence of repetitive regions. The original reads were aligned very poorly against the SOAPdenovo2 and ABySS assemblies, possibly due to misassemblies after chopping the reads into *k*-mers. Moreover, analysis of the sequence datasets using KmerGenie 1.5658 (Chikhi and Medvedev 2014) found no optimal *k*-mer solution.

The assembly was aligned against the GenBank nonredundant nucleotide database using MegaBLAST 2.2.27+ and the National Center for Biotechnology Information contamination screen (Zhang et al. 2000; ). The MegaBLAST results were analyzed in MEGAN 4.70.4 (Huson et al. 2011). Contigs and scaffolds matching contaminants (e.g., Proteobacteria) were removed from the assembly. Genome assembly statistics were calculated using the assemblathon_stats.pl script from the Assemblathon 2 competition (Bradnam et al. 2013). Genome completeness was evaluated using the Core Eukaryotic Gene (CEGs) approach implemented in CEGMA (Parra et al. 2009). The assembly included 47 of 248 complete CEGs (19%) with an additional 53 partial CEGs (21%). As a final test of assembly quality, the known mitochondrial *cox1* and 12S rRNA sequences were identified in the assembly. JR-Assembler had correctly assembled these sequences and placed them in the same scaffold.

### Phylogeographic Analysis of the *Dactylopius coccus* Strain of *Wolbachia* Genome

*Wolbachia* strains exhibit phylogenetic and phylogeographic patterning (Russell et al. 2009). We therefore assembled and analyzed the genome of the *Dactylopius coccus* strain of *Wolbachia* (strain “*w*Coc”) in order to pinpoint the ultimate geographic source of *D. coccus*. The merged cochineal reads were aligned against two complete *Wolbachia* genomes (strains *w*Mel and *w*Pip) (Wu et al. 2004; Klasson et al. 2008) using BWA 0.7.4 (Li and Durbin 2009, 2010). Aligned reads were removed from the sequence pools using a custom script. These reads were used to *de novo* assemble *w*Coc using SOAPdenovo2 (127 bp *k*-mer length, 32 bp minimum mapped read length) (Luo et al. 2012). The *w*Coc genome was then iteratively aligned against the remaining merged reads, the newly aligned sequences were removed from the datasets, and then, the *w*Coc genome was reassembled including the newly removed sequences. This process was repeated until no more reads aligned against the draft genome (two iterations). The final *w*Coc genome sequence totaled 1.13 Mb with an N50 of 1387 bp (TableA2). Previously sequenced *Wolbachia* genomes range in length between 1.0 and 1.5 Mb, indicating that we have sequenced ∽75–100% of the *w*Coc genome.

**Table A2:** *Wolbachia* strain “*w*Coc” genome assembly statistics

Assembly length	1,125,157 bp	Mean sequencing depth	42.9×
N50	1387 bp	L50 count	208
No. scaffolds	1065	Mean scaffold length	1056 bp
Maximum scaffold length:	16,603 bp	Minimum scaffold length	183 bp
Genome %A	33.02%	Genome %T	32.71%
Genome %G	17.18%	Genome %C	17.09%

*Wolbachia* strains are classified primarily by the *ftsZ* gene (Lo et al. 2002). After identification of this gene in *w*Coc, we aligned it against 797 ∽428-bp partial *Wolbachia ftsZ* sequences obtained from GenBank. The analyzed region corresponded to neighboring positions 624,163–624,590 of the *w*Pip genome (Klasson et al. 2008; GenBank accession AM999887.1). A condensed maximum-likelihood tree (100 bootstrap replicates) was then constructed in MEGA 6.06 (Tamura et al. 2013) under a Tamura–Nei (Tamura and Nei 1993) substitution model with invariant sites and a gamma distribution for substitution rates (four gamma categories). *w*Coc fell in clade B with most other insects (Fig.A1). We found little phylogeographic patterning, although it clustered with strains hosted by other scale insects including *Kerria lacca* and *Bemisia tabaci*.
